# Characterization of the aggregates formed during recombinant protein expression in bacteria

**DOI:** 10.1186/1471-2091-6-10

**Published:** 2005-05-31

**Authors:** Andrea Schrödel, Ario de Marco

**Affiliations:** 1 EMBL, Protein Expression Core Facility, Meyerhofstr. 1, D-69117, Heidelberg – Germany

## Abstract

**Background:**

The first aim of the work was to analyze in detail the complexity of the aggregates formed upon overexpression of recombinant proteins in *E. coli*. A sucrose step gradient succeeded in separating aggregate subclasses of a GFP-GST fusion protein with specific biochemical and biophysical features, providing a novel approach for studying recombinant protein aggregates.

**Results:**

The total lysate separated into 4 different fractions whereas only the one with the lowest density was detected when the supernatant recovered after ultracentrifugation was loaded onto the sucrose gradient. The three further aggregate sub-classes were otherwise indistinctly precipitated in the pellet. The distribution of the recombinant protein among the four subclasses was strongly dependent on the DnaK availability, with larger aggregates formed in Dnak^- ^mutants. The aggregation state of the GFP-GST recovered from each of the four fractions was further characterized by examining three independent biochemical parameters. All of them showed an increased complexity of the recombinant protein aggregates starting from the top of the sucrose gradient (lower mass aggregates) to the bottom (larger mass aggregates). These results were also confirmed by electron microscopy analysis of the macro-structure formed by the different aggregates. Large fibrils were rapidly assembled when the recombinant protein was incubated in the presence of cellular extracts, but the GFP-GST fusion purified soon after lysis failed to undergo amyloidation, indicating that other cell components probably participate in the active formation of large aggregates. Finally, we showed that aggregates of lower complexity are more efficiently disaggregated by a combination of molecular chaperones.

**Conclusion:**

An additional analytical tool is now available to investigate the aggregation process and separate subclasses by their mass. It was possible to demonstrate the complexity of the aggregation pattern of a recombinant protein expressed in bacteria and to characterize biochemically the different aggregate subclasses. Furthermore, we have obtained evidence that the cellular environment plays a role in the development of the aggregates and the problem of the artifact generation of aggregates has been discussed using *in vitro *models. Finally, the possibility of separating aggregate fractions with different complexities offers new options for biotechnological strategies aimed at improving the yield of folded and active recombinant proteins.

## Background

The concept of protein aggregation suggests a non-physiological process resulting in the formation of large structures, often chaotic, and in which the proteins have lost their original function/activity. Nevertheless, the collapse of the native conformation can also produce very regular structures, as in the case of amyloid fibrils [1]. Such a process can originate from sensitive protein intermediates during folding as well as from partially denatured proteins that lost their native conformation as a consequence of stress conditions.

Cells possess a sophisticated quality control system to prevent the accumulation of protein aggregates. Molecular chaperones are engaged to promote the correct (re)-folding of misfolded molecules that otherwise undergo protease degradation. Misfolded proteins escaping the quality control may form aggregates that can be trapped in precipitates (aggresome in eukaryotic cells, inclusion bodies in bacteria) to limit their interference with the cell physiology [2]. Inclusion bodies also have a storage function and parts of the trapped proteins are in a dynamic equilibrium with their soluble fraction [3]. Under pathological conditions aggregates develop into structures that hinder the cell functions, as in the case of neuron degenerative diseases.

In bacteria the stress-dependent development of aggregates has been exploited to study the function of the chaperone network. Aggregation has been reversed *in vivo *and the identification of the chaperone combinations necessary for the re-folding of the proteins from aggregates was performed using *in vitro *conditions [4-7]. Nevertheless, the biophysical features of the aggregates have never been investigated. Heat shock is the most studied stress factor but recombinant protein expression can also dramatically modify the cell balance. In fact, the exploitation of highly efficient polymerases increases the rate of protein synthesis so that as much as 50% of the totally accumulated protein can be represented by the recombinant one and the cell folding machinery can become limiting. The optimization of some growth parameters, like the use of low growth temperatures and non-saturating amounts of expression inducer as well as the over-expression of chaperones by means of short heat shock, ethanol stress or recombinant co-expression [8,9], has often improved the yields of recombinant soluble proteins. Nevertheless, in most of the cases part or all of the recombinant protein expressed in bacteria is recovered as precipitates in the inclusion bodies.

Both amorphous and organized inclusion bodies have been isolated [10]. Their composition varies from almost homogeneous to cases in which 50% of the material is represented by contaminants [11,12]. The structural heterogeneity of the inclusion bodies has recently been shown [13,14] and it could be a consequence of the variable aggregation pattern to which a single protein can undergo under different conditions [15]. Proteins trapped in the inclusion bodies can be re-solubilised *in vivo *by impairing the *de novo *protein synthesis because the block of new protein production makes available larger amounts of chaperones and foldases for refolding precipitated proteins [3]. The temporal separation between recombinant expression of chaperones and target proteins has also been successfully used to improve the yield of soluble recombinant proteins [8]. These results suggest a model for which soluble proteins are in a dynamic equilibrium with aggregates. In conclusion, modifications of the cell conditions can modulate the aggregation rate and the protein aggregation process can be reversed by conditions favorable for the folding machinery.

This dynamic view for which proteins can pass from soluble to insoluble and back to soluble state suggests the presence of different degrees of aggregation complexity. Soluble aggregates of recombinant proteins have been described [16,17] and in a recent paper we have shown that the GFP-GST fusion protein expressed in bacteria forms aggregates with an estimated mass ranging from a few hundred kDa to more than 1000 kDa [18]. The separation of the aggregates using a blue native gel electrophoresis followed by SDS-PAGE indicated an almost continuous distribution with few regions of concentrated accumulation. This kind of analysis allows for precise identification of aggregate patterns and comparison among different samples but is not suitable for the further characterization of the aggregates. Therefore, we present here an alternative protocol to separate sub-classes of aggregates using a sucrose step gradient and the results concerning the biophysical organization and biochemical specificities of such aggregates.

## Results and Discussion

### Separation of protein aggregate sub-classes by sucrose step gradient

Preliminary experiments showed that the recombinant GFP-GST produced in bacteria grown at temperature higher than 30°C was mainly recovered in the pellet after ultracentrifugation of the lysates. Nevertheless, decreasing growth temperatures enabled the proportionally inversed recovery of the fusion protein in the supernatant. At 20°C roughly half of the total GFP-GST was in the supernatant (data not shown).

**Figure 1:**
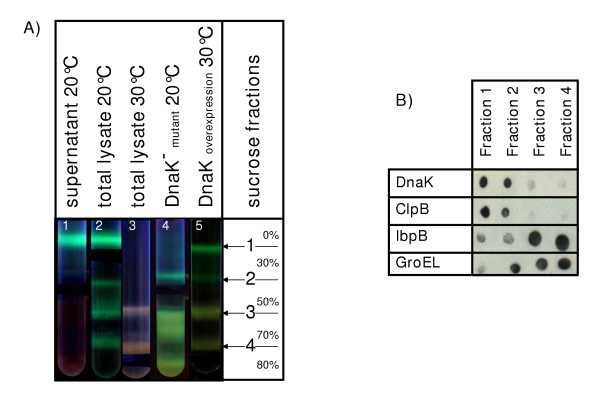
Separation of recombinant GFP-GST fractions by a sucrose step gradient. A) Distribution of the recombinant protein using cell fractions recovered from different bacterial strains and from bacteria grown at different temperatures. Tube number 1 was loaded with the supernatant separated after lysate ultracentrifugation while total lysates were used for the other experiments. B) Dot-blot for the fractions separated by sucrose step gradient. Each fraction was tested with specific antibodies for the chaperones DnaK, ClpB, IbpB and GroEL.

Density gradients have been widely used to separate biological material according to mass. We loaded cell fractions from bacteria induced to express the GFP-GST fusion recombinant protein on a sucrose step gradient to recover sub-classes of aggregates. The fluorescence of GFP-GST simplified the identification of the sucrose concentrations which enabled the separation of the aggregates only at the interface between two different sucrose cushions. Finally, four fractions of GFP-GST were separated when loading a total lysate recovered from bacteria grown at 20°C onto a 0%, 30%, 50%, 70%, 80% sucrose step gradient (Fig. 1A, tube number 2). SDS analysis confirmed that the recombinant GFP-GST was the major protein in all the fractions, however, the co-migrated bacterial proteins were specific for a particular fraction (data not shown). We have already shown that aggregates of GFP-GST can trap other proteins [18] and that chaperones can strongly bind to aggregated recombinant proteins [19]. Dot blot analysis performed using antibodies against the major chaperones showed that DnaK and ClpB were concentrated mostly in the upper gradient fractions -in which the low-density material accumulated- while GroEL and IbpB co-migrated with the larger GFP-GST aggregates (Fig. 1B). These data are in agreement with previous reports that indicated a preferential binding of the different chaperones to aggregates with different degree of complexity [6,7].

The recombinant protein from the four fractions was purified by metal affinity chromatography and both fluorescence and SDS-PAGE analysis indicated that the entire recombinant protein was bound and specifically eluted (data not shown). Protein amount determined by Bradford indicated that, on average, 39% of the total GFP-GST accumulated in the fraction 1, 14%, 22% and 25% in the other three, respectively, from the top to the bottom.

After ultracentrifugation of the lysate, the supernatant was loaded onto the sucrose gradient and the GFP-GST migrated exclusively to the interface between 0% and 30% sucrose (Fig. 1A, tube number 1). We knew from the preliminary experiments that bacteria grown at 30°C produced only insoluble GFP-GST. The fusion protein present in the total lysate from such bacteria was distributed almost exclusively in the fractions 3 and 4 and the fluorescence was almost undetectable (Fig. 1A, tube number 3).

The role of chaperones in limiting the protein aggregation has been widely demonstrated and DnaK has a key role in the chaperone network [4-7]. The sucrose step gradient demonstrated what kind of aggregate pattern modifications occur when the DnaK concentrations vary. No GFP-GST was recovered anymore in the upper fraction when DnaK^- ^mutant bacteria were grown at 20°C and non-fluorescent aggregates largely accumulated in the lower fractions and even on the bottom of the tube (Fig. 1A, tube number 4). In contrast, both soluble GFP-GST and stronger fluorescence were detected after separation of a lysate from bacteria over-expressing DnaK grown at 30°C (Fig. 1A, tube number 5), suggesting that DnaK can improve the GFP-GST stability.

This first set of experiments showed the complexity of the aggregation pattern. In fact, the previously non-characterized insoluble fraction recovered in the pellet was distributed in three classes according to mass and it was possible to separate soluble and insoluble recombinant protein by means of a sucrose gradient. Noteworthy is also the fact that fluorescence can be found in all the four fractions (Fig. 1A), indicating that even in the insoluble aggregates of a larger mass at least part of the trapped recombinant protein conserved a native-like structure. This is in agreement with the report that part of the protein present in the inclusion bodies conserves its secondary structure [20]. Aggregate sub-classes with different complexity and protease resistance have previously been identified in inclusion bodies and also in that case a protein fraction was still active [13,14,21]. In this study, the structural hetereogenity of the proteins trapped in the aggregates is confirmed by our data.

### Biophysical characterization of the GFP-GST fractions separated by the sucrose gradient

The separation of the recombinant GFP-GST on the sucrose gradient is an indication of a mass difference among the aggregates and we wished to confirm these data by size exclusion chromatography (SEC). First, the GFP-GST proteins affinity purified from the four sucrose gradient fractions were dialysed and analysed in the fluorimeter according to the method proposed by Nominé et al. [22], namely the absorbance at 280 and 340 nm was measured and the ratio calculated. This value (aggregation index) indicates the relative aggregation, is quickly determined, and allows the comparison of different fractions of the same protein. Low values indicate a lower aggregation state and our data show that there is a gradient of increasing aggregation from the top fraction to the bottom fractions (Table 1).

**Table 1:** Biophysical characterization of the different aggregate fractions separated by sucrose gradient. The 4 fractions were analysed for their aggregation index, their elution profile using size exclusion chromatography (SEC) and calculating the ratio between aggregated and monodispersed protein, and their binding to the dye ThioflavinT, indicative of amyloid formation. The results refer to one experiment representative of three repetitions.

	**Aggregation index **Abs 280/340 nm	**SEC index **monodispersed/ aggregated protein	**ThioflavinT **Abs 482 nm
Fraction 1	0.38	1.8	4.8
Fraction 2	2.83	0.5	8.8
Fraction 3	3.95	0.4	9.6
Fraction 4	5.96	0.25	13.4

The 4 GFP-GST fractions were also subjected to SEC and the ratio between the areas of the peaks corresponding to the monodispersed and the aggregated protein was calculated (SEC index). Such an index confirmed an increasing state of aggregation from sucrose fraction 1 to 4 (Table 1). Surprisingly, the SEC experiments showed that both aggregated and functional forms of the fusion protein were present in both the three fractions corresponding to the insoluble GFP-GST and the (soluble) fraction 1. Soluble aggregates have been described before and are probably common when fusion proteins are expressed [16,17]. It was not possible to separate monodispersed GFP-GST from soluble aggregates by means of sucrose gradients of decreasing concentrations (data not shown).

We finally tried to characterize the aggregates according to their specific structure. ThioflavinT (ThT) is a dye that preferentially binds to amyloid-like fibrils [23]. We measured an increasing binding when aggregates of higher complexity were used (Table 1). In contrast, there was not significant binding of any aggregate to 8-anilino-1-naphtalenesulfonic acid (ANSA) that has been used as a marker of the amorphous aggregates [24]. This suggests that the aggregates formed by GFP-GST probably have a regular structure involving β-sheets rather than being a chaotic complex held together by hydrophobic interactions. Instead, a micellar organization has been proposed for the soluble aggregates [17,22].

### Aggregate identification by electron microscopy

In the case of the GFP-GST fractions we showed that the degree of amyloidation detected by ThT-binding progressively increased from fraction 1 to fraction 4 (Table 1). The capacity to form fibrils is sequence specific [25] and it seems a generic feature of polypeptide chains [26]. The development into fibrils is characterized by a log phase during which the aggregation seeds are formed followed by a period of rapid growth [27]. Once formed, the fibrils act as aggregation seeds, speeding up the process. Therefore, it could be expected that larger aggregate networks have the possibility to develop faster into structures of higher complexity. In order to test this hypothesis, the GFP-GST from the four sucrose gradient fractions was recovered immediately after centrifugation and mounted for electron microscopy analysis.

**Figure 2:**
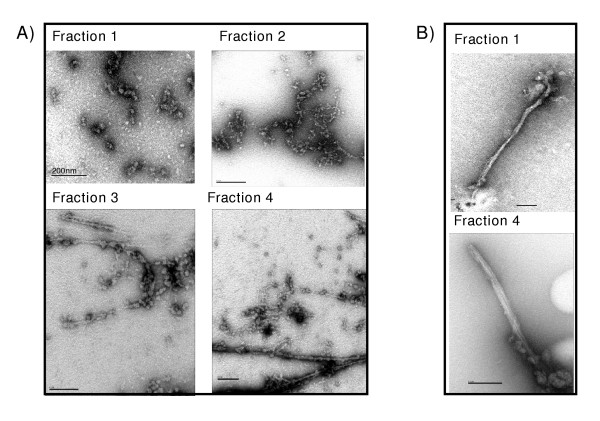
Electron microscopy characterization of GFP-GST macro-aggregates. A) Samples recovered from the 4 aggregation fractions were mounted soon after the sucrose gradient separation and observed by electron microscopy. B) The samples for the electron microscopy grids were from the fractions 1 and 4 recovered after sucrose gradient separation but incubated 24 hours with the co-migrated cell fraction before being mounted.

Some aggregation seeds (20–40 nm in diameter) were visible even when the GFP-GST from the upper fraction was used (Figure 2A, fraction 1). Sort of chains composed by globular elementary structures and measuring several hundreds of nm were observed when GFP-GST from the fraction 2 was exploited (Figure 2A) while protofilaments and higher ordered fibrils [28] longer than 1 μm (Figure 2A) were visible when samples from fractions 3 and 4 were used. Therefore, it was possible to demonstrate the relation between the biochemical indexes used to characterize the aggregation of GFP-GST and the macro-aggregation complexity visible by electron microscopy.

Fibrils are the end product of GFP-GST aggregation but the different classes of aggregates separated by sucrose gradient can be considered as dynamic intermediates that can either develop to larger structures or be reversed into lower-complexity aggregates [29]. Both the initial complexity and the incubation time of polypeptides prone to aggregation are crucial for the building of the aggregates. We wished to demonstrate the importance of these factors in a control experiment. GFP-GST was separated into fractions by sucrose gradient and the fractions 1 and 4 were mounted for electron microscopy only after 24 hours of incubation in the presence of the co-migrated cell components. Both samples raised similar large fibrils (Figure 2B), indicating that the incubation period was sufficient for both, independent of their initial aggregation state, to reach the rapid growth phase that leads to the fibril formation.

This experiment underlines once more the importance of the parameter time in studies dealing with aggregation and questions the meaning of some *in vitro *experiments. In fact, the fibril maturation outside the bacterial cell could have peculiar features. For instance, the lack of space-constrain or limitations in the disaggregation processes could enable the formation of fibrils the length of which are difficultly compatible with the size of *E. coli *cells (Figure 2B). The experiments described in the two last paragraphs will show the impact of cell components in promoting aggregation and disaggregation.

Finally, the presence of aggregation seeds smaller than 40 nm in diameter shows that it is not possible to discriminate between soluble and aggregated fractions by the use of simplified methods in high-throughput protocols as, for instance, the exploitation of a 0.65 μm pore size filter [30].

### Is the aggregation of GFP-GST actively supported?

**Figure 3:**
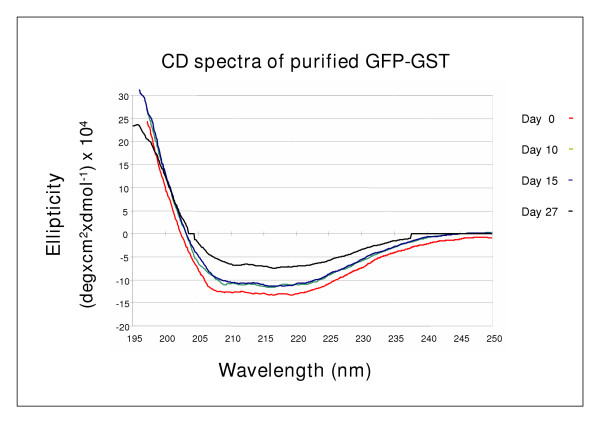
Circular dichroism spectra of GFP-GST. Protein purified by metal affinity from the supernatant obtained after lysate ultracentrifugation was directly analysed (day 0) or incubated at room temperature before the collection of further spectra the days 10, 15, and 27.

In the previous experiments we showed that even the moderately aggregated GFP-GST recovered from the upper fraction of the sucrose gradient could form fibrils if the sample was incubated with the cell fraction for at least 1 day before it was prepared for the electron microscopy analysis. In a recent paper it was claimed that bacterial chaperones play an active role in the formation of the aggregates [31]. The possible participation of cell components in catalyzing the GFP-GST fibril formation was investigated in a control experiment. The process of aggregate maturation of the soluble recombinant protein in the presence of other cell components was limited to 1 hour performing the affinity purification of the GFP-GST immediately after lysis to avoid a seeding process during the 15 hour centrifugation of the cell components upon the sucrose gradient. The sample was incubated at room temperature for 4 weeks and the modifications of the secondary structure were monitored by CD while corresponding samples were mounted for electron microscopy. No significant modification was observed in the first two weeks and a slight increase of the β-sheet content was measured only after 4 weeks (Figure 3). The use of different protein concentrations and the addition of sucrose to the proteins did not modify the pattern and no detectable aggregate was observed at the electron microscopy using the corresponding samples (data not shown).

Therefore, these results strongly suggest that the co-presence of other molecules is necessary to trigger the process of regular aggregation of the recombinant protein, probably by facilitating the formation of aggregation seeds. Chaperones can play a role in the aggresome formation [32] and GroEL has been claimed to be actively involved in bacterial inclusion body formation [31]. Our data can only confirm that GroEL co-migrates with the aggregates of larger mass (Fig. 1B). Finally, we are looking for an analytical method to determine if the process of cell lysis is crucial for the development of the aggregates.

### Aggregate complexity and re-folding

Both *in vivo *and *in vitro *experiments illustrated the co-operative action of chaperone networks in disaggregating misfolded proteins [4-7] but the features of the real aggregates that are the target of the chaperones in the cells have never been investigated. We used the aggregates from fractions 3 and 4 to test if they could be a substrate for chaperone-dependent refolding and if the different structure complexity had a role on the refolding kinetic.

**Figure 4:**
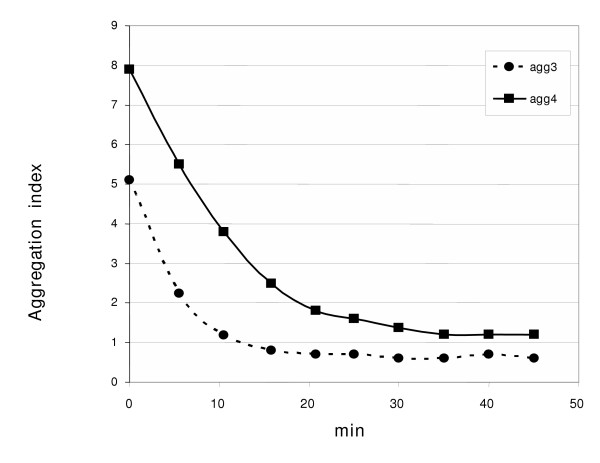
Chaperone-dependent *in vitro *disaggregation. Purified GFP-GST aggregates recovered from the fractions 3 and 4 of the sucrose gradient were incubated in the presence of an equimolar mixture of DnaK, DnaJ, GrpE, and ClpB in the presence of a system constantly providing ATP. The aggregation index was repeatedly measured during a 45 min incubation.

An equimolar combination of DnaK, DnaJ, GrpE, and ClpB [6] quickly disaggregated the large precipitates (Figure 4). Specifically, the complexity of the aggregates from fraction 3 was reduced in a faster and more efficient way. In fact, the aggregation index dropped by half in only 4 min while it took 10 min in the case of the aggregates from fraction 4. Furthermore, there was a higher residual aggregation: the aggregation indexes measured were 1.2 and 0.7 for the aggregates from fractions 4 and 3, respectively. In comparison, the GFP-GST from fraction 1 scored 0.38 (Table 1). The addition of equimolar amounts of BSA to the aggregates in absence of chaperones had no disaggregation effect.

The preferential disaggregation of subclasses of aggregates with lower complexity observed *in vitro *is reminiscent of previous works indicating that specific subclasses of the proteins trapped in the inclusion bodies are preferentially refolded under physiological conditions [3,13] and that the reversibility is increasingly difficult and dependent on the size of the aggregates [29]. The limit of this experiment is that it is difficult to scale up and the small amount of the protein used was insufficient for undertaking further biophysical analysis. The aggregation index gives only relative values and, therefore, we can state that the degree of aggregation decreased but cannot conclude that the disaggregated protein was also correctly folded. Nevertheless, the results suggest that it would be of biotechnological interest to separate the aggregate subclasses and use the lower complexity aggregates in refolding protocols.

## Conclusion

There is increasing evidence that aggregates are heterogeneous in size and complexity [2,12-16,26]. The aggresomes are actively built in eukaryotic cells and the physiological meaning of the process would be the packing of disorganized aggregates that could interfere with the normal cell functions by non-specifically binding to other cell components [33,34]. The possibility to recover functional proteins from the insoluble aggregates [3] would indicate that at least in bacteria they can function as a reserve in dynamic equilibrium with soluble fractions.

**Figure 5:**
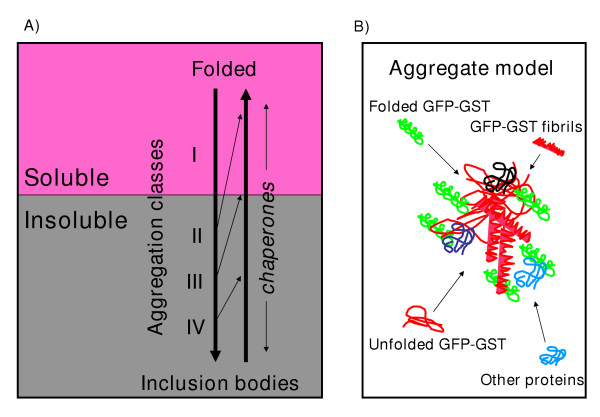
Schematic representation of the aggregation. A) Dynamic of the aggregation. GFP-GST aggregates progressively form both soluble and insoluble aggregates. Chaperone activity can reverse the process of aggregation in a way that is inversely proportional to the degree of complexity reached by the aggregates and could also play a role in the aggregate maturation towards more structured complexes. B) Aggregate model. The aggregation of GFP-GST probably starts with misfolded single proteins that collapse into pre-fibrillar structures. These catalyze the aggregation of new molecules to form larger amyloid fibrils. In the initial phases, the co-presence of molecules with different degree of misfolding and amyloidation seems apparent. Pre-fibrils could form the core of the aggregation seeds to which partially misfolded GFP-GST molecules bind. Some of these still conserve a native-like structure compatible with fluorescence functionality. The aggregation nets can trap other proteins in a probably non-specific manner.

The expression of recombinant proteins is a stress factor because they compete for energy and substrates with native expression and can interfere with the normal metabolism by forming aggregates, both in prokaryotic and eukaryotic cells [2,34]. The possibility to store the excess of misfolded recombinant protein could be a way to get rid of dangerous aggregating material when misfolded proteins escaped the quality control of chaperones and proteases [2]. The cellular mechanisms that favor the generation of amyloids (Figure 2) might also be useful in preventing amorphous aggregates in non-specifically trapping native proteins [18]. The aggregate organization would consider an aggregate mash that grow from small entities towards larger insoluble structures [34] composed by a core of protease-resistant fibrils [13,14], homologous proteins at different levels of misfolding and some heterologous and non-specifically trapped proteins [18] (Figure 5B).

In this paper we present data supporting the idea of a progressive maturation of recombinant GFP-GST aggregates into amyloid fibrils. Furthermore, it seems that the process is facilitated by some other cell components since the fibril maturation was extremely slower when the recombinant protein was separated from the other cell components soon after the lysis (Fig. 3). For instance, GroEL has been reported having an active role in inclusion body formation [31] and specifically co-migrate with the larger aggregates could (Fig. 1B). Conversely, the combination of DnaK, DnaJ, GrpE and ClpB could disaggregate large insoluble structures (Figures 4 and 5A).

It seems that the aggregation process of recombinant proteins is extremely more complicated than normally accepted and our separation protocol turned out to be a useful tool for characterizing the aggregates. Furthermore, such an aggregation process shares many features with the maturation of pathological amyloids in eukaryotic cells and, therefore, the bacterial system -experimentally easy to modify- would be considered as a model to integrate the results obtained using *in vitro *systems and to study the impact of chemical and biophysical parameters on the aggregation development. We simplified the work by using a fluorescent construct but any protein for which antibodies are available could be used for following the aggregation development.

## Methods

### Cell culture and protein preparation

A fusion construct His-GST-GFP cloned in a Gateway destination vector (Invitrogen, kindly provided by D. Waugh) was transformed and expressed in the following bacterial strains: BL 21 (DE3), BL 21 (DE3) RIL codon plus, GK2 (*dnak*^-^), BL 21 (DL3) co-expressing the chaperone combinations GroELS and GroELS/DnaK/DnaJ/GrpE/ClpB, respectively (kindly provided by B. Bukau). Bacteria were grown at 37°C until the OD_600 _reached 0.4, then the cultures were adapted to different temperatures (20°C, 25°C, 30°C, 37°C), induced at an OD_600 _of 0.6 with 0.1 mM IPTG and grown for further 20 h. The bacteria were pelleted by centrifugation (6000 g × 15 min), washed in 10 mL of PBS and finally stored at -20°C.

The pellet was resuspended in 10 mL of lysis-buffer (50 mM potassium phosphate buffer, pH 7.8, 0.5 M NaCl, 5 mM MgCl_2_, 1 mg/mL lysozyme, 10 μg/mL DNase), sonicated in a water bath (Branson 200) for 5 min and the lysate was incubated for 30 min on a shaker at room temperature. The supernatant was recovered after ultracentrifugation (35 min at 150000 × g).

Fractions from sucrose gradients were recovered using a bent Pasteur pipette and affinity purified using a HiTrap chelating affinity column (Amersham Biosciences) pre-equilibrated with 20 mM Tris HCl, pH 7.8, 500 mM NaCl, 15 mM imidazole. The His-tagged recombinant protein was eluted in 20 mM Tris, pH 7.8, 125 mM NaCl, and 250 mM imidazole. Protein quantification was based on the absorbance at 280 nm.

### Sucrose gradients and gel filtration

Total cell lysates or supernatants from ultracentrifugation of total cell lysates (1 mL) were loaded onto 14 × 95 mm Ultra-Clear centrifuge tubes (Beckman) prepared with a step gradient formed by four layers of 20 mM TrisHCl buffer, pH 8, containing 80%, 70%, 50%, 30%, and 0% sucrose, respectively. The tubes were centrifuged 15 hours at 180,000 × g at 4°C using a SW40Ti rotor and a L-70 Beckman ultracentrifuge. The protein fractions were recovered from the interfaces between two sucrose layers, affinity purified as described above and used for further analysis. The samples for gel filtration were concentrated and the buffer replaced with 50 mM TrisHCl, pH8.0, 150 mM NaCl using a Vivapore concentrator (Vivascience) and then separated by gel filtration using a Superose 12 HR 10/30 column (Amersham).

### Bioanalytical assays

The aggregation rate of the proteins was analysed according to Nominé et al. [22] using an AB2 Luminescence Spectrometer (Aminco Bowman Series 2) equipped with SLM 4 software. The excitation was induced at 280 nm and the emission scan was recovered between 260 and 400 nm.

Amyloid aggregates were estimated according to their binding to the specific dye thioflavin-T (ThT), as described by LeVine [23], and protein surface hydrophobicity was determined using the fluorescent probe 8-anilino-1-naphtalenesulfonic acid (ANSA) [24].

Circular dichroism (CD) spectra were recorded between 250 and 190 nm using suprasil precision cells (Hellma) and a Jasco J-710 instrument.

### Western and dot blotting

Western blots were performed as previously described [18] using anti-GST primary antibodies. For dot blotting the proteins were transferred onto a PVDF membrane using a Bio-Rad Criterion blotter. The primary rabbit antibodies were a gift from Dr. Bukau and were purified from sera using Protein G Plus/Protein A Agarose (Oncogene) to minimize the background. Peroxidase-conjugated secondary antibodies for chemioluminescent detection were purchased from Dianova and the detection performed using the SuperSignal^® ^West Femto Maximum Sensitivity Substrate (Pierce), following the supplier's instructions. Blots were used repeatedly by effectively removing the antigen-antibody interaction using the Western Blot Recycling it (Alpha Diagnostic Int.).

### Sample preparation for electron microscopy

Protein samples were purified by affinity chromatography and equal amounts fixed by using the "single-droplet" parafilm protocol. 5 μL of each protein sample were pipetted on a grid (Agar Scientific) and incubated 1 min at room temperature. Excess fluid was removed using filter paper, the unbound protein was washed and the grids were placed on a 50 μL drop of 1% uranyl acetate with the section side downwards. Finally, the grids were dried, placed in the grid-chamber and stored in desiccators before the samples were observed with a CM120 BioTwin electron microscope (Philips).

### *In vitro *re-folding assay

The conditions for the chaperone-dependent disaggregation of GST-GFP *in vitro *were chosen according to Mogk et al. [35] and the process was monitored using the fluorimetric assay described above [22]. 1 μM of aggregated protein was resuspended in 50 mM Tris HCl, pH 7.5, 20 mM MgCl_2_, 150 mM KCl, 2 mM DTT, in the presence of 1 μM ClpB, 1 μM DnaK, 0.2 μM DnaJ, 0.1 μM GrpE, 3 mM phosphoenolpyruvate, and 20 ng/mL of pyruvate kinase. The reaction was started by the addition of 2 mM NaATP.

## Authors' contributions

Andrea Schrödel performed all the experiments at least once. Ario de Marco conceived the study, repeated some of the experiments and wrote the manuscript.

## References

[B1] Harper JD, Lansbury PT (1997). Models of amyloyd seeding in Alzheimer's disease and Scrapie. Annu Rev Biochem.

[B2] Kopito RR (2000). Aggresomes, inclusion bodies and protein aggregation. Trends Cell Biol.

[B3] Carrió MM, Villaverde A (2001). Protein aggregation as bacterial inclusion bodies is reversible. FEBS Lett.

[B4] Veinger L, Diamant S, Buchner J, Goloubinoff P (1998). The small heat-shock protein IbpB from *Escherichia coli *stabilizes stress-denatured proteins for subsequent refolding by a multichaperone network. J Biol Chem.

[B5] Mogk A, Tomoyasu T, Goloubinoff P, Rüdiger S, Röder D, Langen H, Bukau B (1999). Identification of thermolabile *Escherichia coli *proteins: prevention and reversion of aggregation by DnaK and ClpB. EMBO J.

[B6] Goloubinoff P, Mogk A, Ben Zvi AP, Tomoyasu T, Bukau B (1999). Sequential mechanism of solubilization and refolding of stable protein aggregates by a bichaperone network. Proc Natl Acad Sci USA.

[B7] Mogk A, Deuerling E, Vorderwulbecke S, Vierling E, Bukau B (2003). Small heat shock proteins, ClpB and the DnaK system form a functional triade in reversing protein aggregation. Mol Microbiol.

[B8] de Marco A, De Marco V (2004). Bacteria co-transformed with recombinant proteins and chaperones cloned in independent plasmids are suitable for expression tuning. J Biotechnol.

[B9] Steczko J, Donoho GA, Dixon JE, Sugimoto T, Axelrod B (1991). Effect of ethanol and low-temperature culture on expression of soybean lipoxygenase L-1 in *Escherichia coli*. Prot Expr Purif.

[B10] Bowden GA, Peredes AM, Georgiou G (1991). Structure and morphology of protein inclusion bodies in *Escherichia coli*. Biotechnol (NY).

[B11] Valax P, Georgiou G (1993). Molecular characterization of beta-lactamase inclusion bodies produced in *Escherichia coli*. 1. Composition. Biotechnol Prog.

[B12] Speed MA, Wang DIC, King J (1996). Specific aggregation of partially folded polypeptide chains: the molecular basis of inclusion body composition. Nat Biotechnol.

[B13] Carrió MM, Cubarsi R, Villaverde A (2000). Fine architecture of bacterial inclusion bodies. FEBS Lett.

[B14] Carrió MM, Gonzalez-Montalban N, Vera A, Villaverde A, Ventura S (2005). Amyloid properties of bacterial inclusion bodies. J Mol Biol.

[B15] Ben Zvi AP, Goloubinoff P (2002). Proteinaceous infectious behavior in non-pathogenic proteins is controlled by molecular chaperones. J Biol Chem.

[B16] Sachdev D, Chirgwin JM (1999). Properties of soluble fusions between mammalian aspartic proteases and bacterial maltose-binding protein. Biochem J.

[B17] Nominé Y, Ristriani T, Laurent C, Lefevre J-F, Weiss E, Travé G (2001). Formation of soluble inclusion bodies by HPV E6 oncoprotein fused to maltose-binding protein. Prot Expr Purif.

[B18] Stegemann J, Ventzki R, Schrödel A, de Marco A (2005). Comparative analysis of protein aggregates by blue native electrophoresis and subsequent SDS-PAGE in a three-dimensional geometry gel. Proteomics.

[B19] Carrió MM, Corchero JL, Villaverde A (1998). Dynamics of *in vivo *protein aggregation: building inclusion bodies in recombinant bacteria. FEMS Microbiol Lett.

[B20] Oberg K, Chrunyk BA, Wetzel R, Fink AL (1994). Native-like secondary structure in interleukin-1β inclusion bodies by attenuated total reflectance FT-IR. Biochemistry.

[B21] Tokatlidis K, Dhurjati P, Millet J, Beguin P, Albert JP (1991). High activity of inclusion bodies formed in *Escherichia coli *overproducing *Clostridium thermocellum *endoglucanase D. FEBS Lett.

[B22] Nominé Y, Ristriani T, Laurent C, Lefevre J-F, Weiss E, Travé G (2001). A strategy for optimizing the monodispersity of fusion proteins: application to purification of recombinant HPV E6 oncoprotein. Prot Engineer.

[B23] LeVine H (1999). Quantification of beta-sheet amyloid fibril structures with ThioflavinT. Methods Enzymol.

[B24] Busby TF, Atha DH, Ingham KC (1981). Thermal denaturation of antithrombin III. Stabilization by heparin and lyotropic anions. J Biol Chem.

[B25] Linding R, Schymkowitz J, Rousseau F, Diella F, Serrano L (2004). A comparative study of the relationship between protein structure and beta-aggregation in globular and intrinsically disordered proteins. J Mol Biol.

[B26] Dobson CM (2003). Protein folding and misfolding. Nature.

[B27] Caughey B, Lansbury PTJr (2003). Protofibrils, pores, fibrils, and neurodegeneration: separating the responsible protein aggregates from the innocent bystanders. Annu Rev Neurosci.

[B28] Holm Nielsen E, Nybo M, Svehag S-E (1999). Electron microscopy of prefibrillar structures and amyloid fibrils. Methods Enzymol.

[B29] Calamai M, Canale C, Relini A, Stefani M, Chiti F, Dobson CM (2005). Reversal of protein aggregation provides evidence for multiple aggregated states. J Mol Biol.

[B30] Dyson MR, Shadbolt SP, Vincent KJ, Perera RL, McCafferty J (2004). Production of soluble mammalian proteins in Escherichia coli: identification of protein features that correlate with successful expression. BMC Biotechnology.

[B31] Carrió MM, Villaverde A (2003). Role of molecular chaperones in inclusion body formation. FEBS Lett.

[B32] Garcia-Mata R, Bebok Z, Sorscher EJ, Sztul ES (1999). Characterization and dynamics of aggresome formation by a cytosolic GFP chimera. J Cell Biol.

[B33] Johnston JA, Wand CL, Kopito RR (1998). Aggresomes: a cellular response to misfolded proteins. J Cell Biol.

[B34] Stefani M, Dobson CM (2003). Protein aggregation and aggregate toxicity: new insights into ptotein folding, misfolding diseases and biological evolution. J Mol Med.

[B35] Mogk A, Schlieker C, Friedrich KL, Schönfeld H-J, Vierling E, Bukau B (2003). Refolding of substrates bound to small Hsps relies on a disaggregation reaction mediated most efficiently by ClpB/DnaK. J Biol Chem.

